# Diversified Soil Types Differentially Regulated the Peanut (*Arachis hydropoaea* L.) Growth and Rhizosphere Bacterial Community Structure

**DOI:** 10.3390/plants14081169

**Published:** 2025-04-09

**Authors:** Wenfei Lan, Hong Ding, Zhimeng Zhang, Fan Li, Hao Feng, Qing Guo, Feifei Qin, Guanchu Zhang, Manlin Xu, Yang Xu

**Affiliations:** 1 Shandong Peanut Research Institute, Qingdao 266100, China; lwf200008@163.com (W.L.); dingpeanut@163.com (H.D.); qinhdao@126.com (Z.Z.); 15949788904@163.com (F.L.); ben0917@163.com (H.F.); jone007@126.com (Q.G.); jialing_300@163.com (F.Q.); guanchuzhang@126.com (G.Z.); xumanlin@126.com (M.X.); 2 College of Agriculture, Xinjiang Agricultural University, Urumqi 830052, China

**Keywords:** peanut (*Arachis hydropoaea* L.), soil type, metagenome, rhizosphere bacterial community, saline–alkali soil, acidic soil

## Abstract

Peanut (*Arachis hydropoaea* L.) demonstrates a prominent adaptability to diverse soil types. However, the specific effects of soil types on peanut growth and bacterial communities remain elusive. This study conducted a thorough examination of the agronomic traits, the corresponding physicochemical properties, and bacterial structure of rhizosphere soil in acidic (AT), neutral (NT), and saline–alkali (ST) soils, elucidating the internal relationship between soil type and peanut yield. Our results showed that different soil types exhibited significant differences in peanut yield, with ST demonstrating the lowest yield per plant, showing an 85.05% reduction compared to NT. Furthermore, available phosphorus content, urease, and invertase activities were substantially reduced in both ST and AT, particularly in ST by 95.35%, 38.57%, and 62.54%, respectively. Meanwhile, metagenomic sequencing unveiled a notable decline in *Bradyrhizobium* and *Streptomyces* in these soils, which is crucial for soil improvement. Further metabolic pathway analysis revealed that the reduction in pathways related to soil remediation, fertility improvement, and stress response in AT and ST may lead to slower peanut growth. In conclusion, peanuts cultivated in acidic and saline–alkali soils can increase yield via implementing soil management practices such as improving soil quality and refining micro-environments. Our study provides practical applications for enhancing peanut yield in low- to medium-yield fields.

## 1. Introduction

Peanut (*Arachis hydropoaea* L.), one of the important oil and economic crops widely cultivated globally, is not only influenced by its genetic characteristics and cultivation management practices but is also significantly constrained by soil environments [1,2]. In agricultural production, soil constraints such as salinization, heavy metal pollution, nutrient imbalance, and continuous cropping obstacles pose severe threats to peanut yield and quality. Different soil types have significant effects on peanut agronomic traits, photosynthesis, chlorophyll content, yield, and soil bacterial community structure [3,4]. Saline–alkali soils, in particular, due to high salt content and pH as well as low nutrient content, cause calcium, magnesium, and other heavy metals in the soil to form insoluble precipitates with phosphate and trace elements, inhibiting nutrient absorption and affecting root growth and ultimately leading to issues such as yellowing leaves, root rot, and plant death [5]. Additionally, saline–alkali stress affects the synthesis and ratio of fatty acids in peanut kernels, thereby affecting peanut quality and yield [6,7,8,9,10]. In acidic soils, high aluminum ion concentrations can affect peanut seed germination, resulting in reduced germination rates and shortened roots. Moreover, the solubility of phosphorus decreases, weakening peanut’s ability to absorb phosphorus; peanut pegs face difficulties in penetrating the soil, leading to poor pod development and increased susceptibility to pod rot, thus affecting peanut yield and quality [4,11,12,13]. In summary, saline–alkali and acidic soils are two major soil types that severely impact peanut production.

The rhizosphere is a key area where soil microorganisms interact with plant roots. The roots provide habitat and nutrients for microorganisms, while the microorganisms reciprocate by decomposing organic matter, releasing nutrients, and producing disease- and pest-resistant substances to influence plant growth and enhance plant stress resistance [14,15,16,17]. Various tillage practices, soil types, and crop growth stages all exert specific influences on the bacterial communities associated with crop roots, among which soil types are an important regulatory factor [18,19,20]. The physicochemical properties and nutrient contents of the various soil types exhibit profound differences, which may prompt adaptive transformations within the bacterial community of the plant rhizosphere, ultimately influencing the plant’s growth performance and its ability to withstand stress [21]. Clay is linked to a reduction in the diversity indices of bacterial communities, along with alterations in the composition of Proteobacteria and Actinobacteria [22]. Chloroflexi is relatively abundant in acidic red soils, while Proteobacteria is more abundant in black soils with a high organic content [23,24]. In acidic soils, Cyanobacteria is more abundant; in neutral soils, the abundant phyla include Proteobacteria, Actinobacteria, Chloroflexi, and Acidobacteria, and in alkaline soils, the phyla that dominate include Proteobacteria, Gemmatimonadetes, Nitrospirae, Bacteroidetes, and Firmicutes [25,26,27,28]. In saline–alkali soils, the rhizosphere soil is enriched with bacterial communities dominated by the *Pseudomonas*, and compared to neutral soils, the number of Cyanobacteria and Proteobacteria is increased, while the number of Acidobacteria decreases [2,29,30]. This indicates that the relative abundance of rhizosphere microorganisms varies across different soil types.

Given that most bacterial species are non-culturable without prior understanding of their characteristics, the use of metagenomics technology offers an exceptional opportunity to identify the rhizosphere bacteriome [31]. Research on peanut rhizosphere bacterial communities has become increasingly in-depth with metagenomics sequencing. However, there remains a lack of comprehensive analysis regarding peanut agronomic indicators and bacterial community diversity in various soil types, such as saline–alkali and acidic soils. Physicochemical properties and soil enzyme activity analysis can serve as valuable complements to the metagenomics to offer a comprehensive display of the distinctive characteristic of rhizosphere soil micro-environment. Therefore, this study endeavors to employ metagenomic sequencing technology and physicochemical factor detection to conduct a thorough analysis of peanut rhizosphere soil characteristics across different soil types.

Our objective is to uncover the disparities in structure and function in the rhizosphere micro-environment across diverse soil types and to investigate the implications of these differences on peanut growth and development. Ultimately, this research aims to provide scientific evidence and practical applications for increasing peanut yield in low- to medium-yield fields.

## 2. Results

### 2.1. Effects of Different Soil Types on Peanut Agronomic Traits

**Figure 1:**
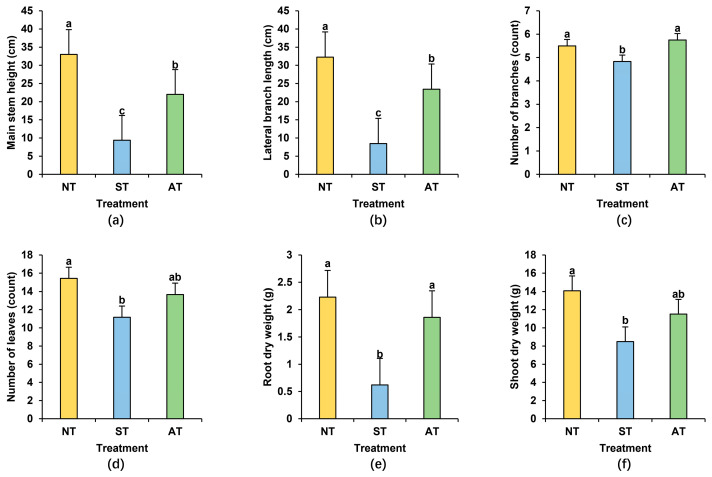
Effects of different soil types on peanut agronomic traits (**a**–**f**): main stem height (**a**), lateral branch length (**b**), number of branches (**c**), number of leaves (**d**), root dry weight (**e**), and shoot dry weight (**f**) of peanuts cultivated in different soil types. Different lowercase letters indicate significant difference at *p* < 0.05 among the treatments.

To investigate the agronomic traits of peanuts cultivated in different soil types, including acidic soil (AT), neutral soil (NT), and saline–alkali soil (ST), we conducted a statistical analysis of physiological indicators of peanuts grown in these soil conditions. The results revealed the consistent trend NT > AT > ST for main stem height, lateral branch length, leaf number, root dry weight, and shoot dry weight of the peanuts (Figure 1). Notably, the differences in main stem height and lateral branch length were particularly prominent: peanuts grown in ST exhibited a 71.58% reduction in main stem height and a 73.77% decrease in lateral branch length, while those in AT showed a more modest decline of 33.33% and 27.38%, respectively (Figure 1a,b). Remarkably, all indicators for peanuts cultivated in ST were significantly lower than those in NT, with decreases of 12.18%, 27.56%, 72.20%, and 39.70% in the number of branches, number of leaves, root dry weight, and shoot dry weight, respectively (Figure 1c–f). In contrast, these four agronomic indicators exhibited no discernible difference in AT when compared with those in NT (Figure 1c–f). In conclusion, ST exerted a more pronounced influence on the agronomic traits of peanuts compared to AT, demonstrating that it is more detrimental to peanut growth.

### 2.2. Differential Peanut Yield in Diverse Soil Types

**Figure 2:**
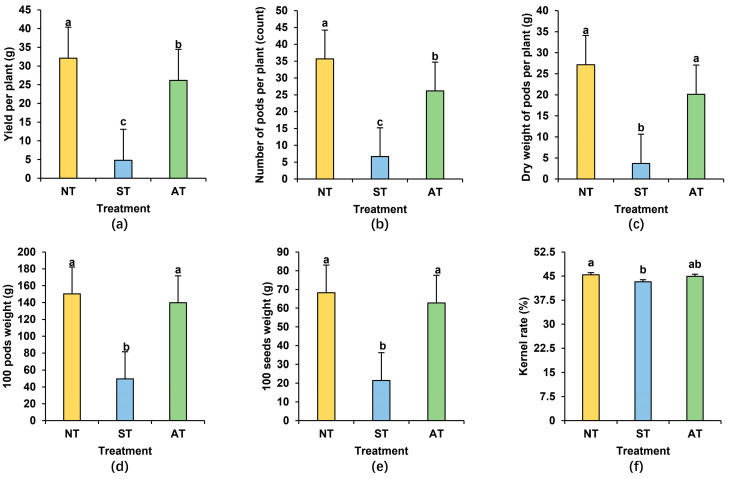
The yield components of peanuts cultivated in different soil types (**a**–**f**): yield per plant (**a**), number of pods per plant (**b**), dry weight of pods per plant (**c**), 100 pods weight (**d**), 100 seeds weight (**e**), and kernel rate (**f**) of peanuts cultivated in different soil types. Different lowercase letters indicate significant difference at *p* < 0.05 among the treatments.

Through a thorough assessment of peanut yield components across various soil types, we discovered that ST demonstrated the lowest yield per plant, with AT following behind (Figure 2a). Specifically, ST led to an 85.05% decrease in yield per plant compared with NT by reducing the number of pods per plant, dry weight of pods per plant, 100 pods weight, 100 seeds weight, and kernel rate by 81.30%, 86.41%, 67.01%, 68.01%, and 4.85%, respectively (Figure 2b–f). Peanut yield components in AT showed slightly lower downward trends compared with those in NT, with yield per plant decreasing by 18.51% and the number of pods per plant declining by 26.63% (Figure 2a,b). It is apparent that peanuts are more suitable for cultivation in acidic soil compared to saline–alkali soil.

### 2.3. Rhizosphere Soil Physicochemical Properties Analysis in Different Soil Types

**Figure 3:**
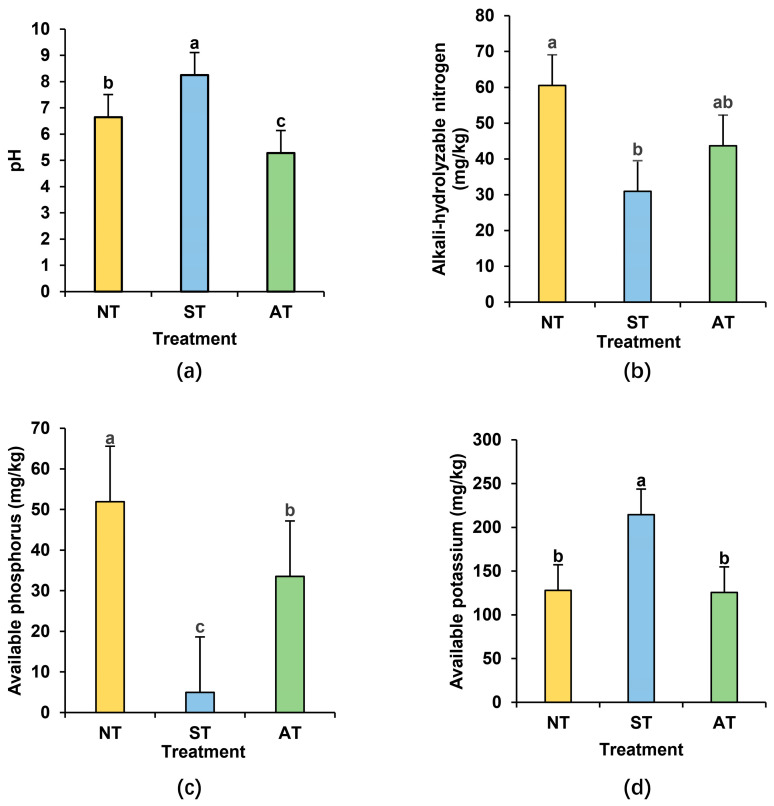
Peanut rhizosphere soil physicochemical properties in different soil types (**a**–**d**): the pH (**a**), content of alkali-hydrolyzable nitrogen (**b**), available phosphorus (**c**), and available potassium (**d**) in peanut rhizosphere soil of different soil types. Different lowercase letters indicate significant difference at *p* < 0.05 among the treatments.

Regarding the rhizosphere soil properties, ST has a pH of 8.25, clearly indicating its alkaline nature (Figure 3a). Compared with NT, the contents of alkali-hydrolyzable nitrogen and available phosphorus in ST significantly declined, with reductions of 50.00% and 95.35%, respectively (Figure 3b,c). Conversely, the content of available potassium showed contrary trends in ST, increasing by 67.60% compared with that in NT (Figure 3d). On the other hand, AT has a pH of 5.28, indicating acidity (Figure 3a). The contents of alkali-hydrolyzable nitrogen and available potassium in AT remain basically unchanged compared with those in NT, while the content of available phosphorus is reduced by 58.21% (Figure 3b–d). Saline–alkali soil, which contains lower amounts of alkali-hydrolyzable nitrogen and available phosphorus compared to acidic soil, may pose more adverse conditions for peanut growth.

### 2.4. Soil Types Altered Rhizosphere Soil Enzyme Activities

**Figure 4:**
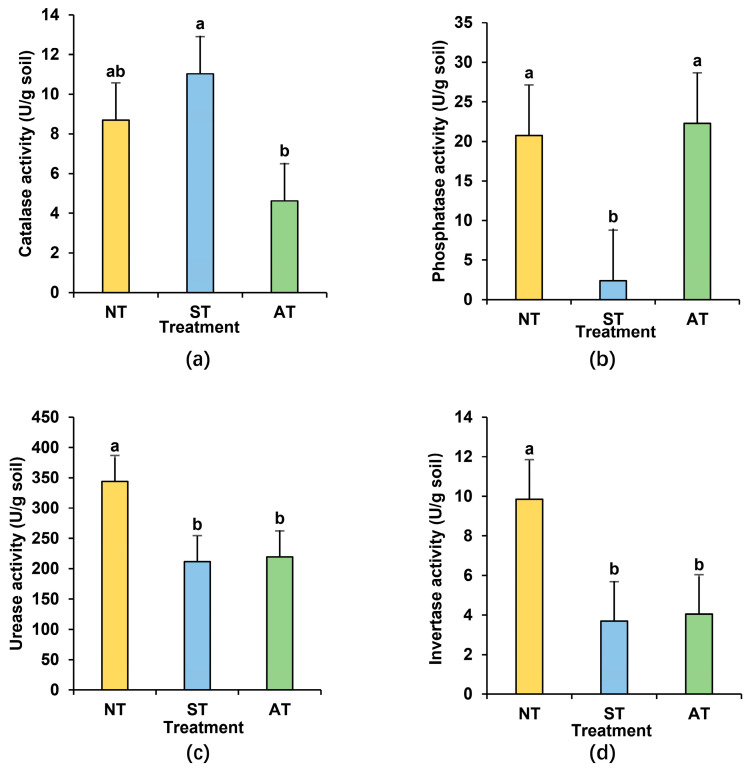
Soil enzyme activities in peanut rhizosphere soil under different soil types (**a**–**d**): catalase activity (**a**), phosphatase activity (**b**), urease activity (**c**), and invertase activity (**d**) in peanut rhizosphere soil of different soil types. Different lowercase letters indicate significant difference at *p* < 0.05 among the treatments.

To explore how soil exogenous enzyme activities vary with the changes in the physicochemical properties across different rhizosphere soils, we conducted measurements of catalase, phosphatase, urease, and invertase activities in diverse soil types (Figure 4). The findings showed that, with the exception of soil catalase activity, which exhibited comparable levels, the other three soil enzyme activities all declined in ST (Figure 4a–d). Specifically, the average phosphatase, urease, and invertase activities in ST decreased markedly by 88.53%, 38.57%, and 62.54%, respectively, when compared with those in NT (Figure 4b–d). In AT, only urease and invertase activities were significantly lower than those in NT, with reductions of 36.19% and 58.99%, respectively (Figure 4c,d). In conclusion, different soil types demonstrate considerable differences in their impact on rhizosphere soil enzyme activities, with ST exerting a more severe decline on enzyme activities than AT, potentially generating an unfavorable condition for peanut growth.

### 2.5. Alpha and Beta Diversity of the Peanut Rhizosphere Bacterial Community in Different Soil Types

Rhizosphere microorganisms and their community structure constitute a pivotal factor influencing root development. To delve into the difference of diverse soil types on the composition of the peanut rhizosphere bacterial communities, we conducted metagenome sequencing analysis on rhizospheres sampled from the above distinct soil types. From these, we garnered an average of 44,291,149, 46,462,142, and 46,296,365 raw reads, respectively, translating into approximately 6687.96 Mbp, 7015.78 Mbp, and 6990.75 Mbp of valuable data (Table S1). Subsequently, we assembled the clean reads (Table S2), yielding an average of 469,539.67, 300,751.00, and 467,742.33 contigs for NT, ST, and AT, respectively. Upon predicting open reading frames (ORFs) within these contigs, we found that NT, ST, and AT generated 611,719.33, 346,509.30, and 585,302.00 ORFs, respectively. After removing redundancy from the ORFs, we obtained a substantial number of non-redundant genes: 1,314,285.00 for NT, 586,774.70 for ST, and 1,229,599.00 for AT. These results underscore the substantial volume of metagenome sequencing data obtained, which provides comprehensive coverage of the bacterial community to meet the prerequisites for subsequent in-depth analysis.

Alpha diversity is a comprehensive index reflecting richness and evenness [32]. We systematically analyzed the ACE, Shannon, and Simpson indices of the peanut rhizosphere bacterial community under different soils at the phylum, class, order, family, and genus levels. Analysis of the bacterial richness indices ace showed that the peanut rhizosphere bacterial richness under ST and AT was significantly lower than that under NT at the five levels. However, there were no significant differences in the bacterial evenness for Shannon and Simpson indices (Table 1). The results indicate that the bacterial richness under ST and AT is significantly lower than that of the control, but no significant difference is discernible in terms of bacterial evenness.

**Figure 5:**
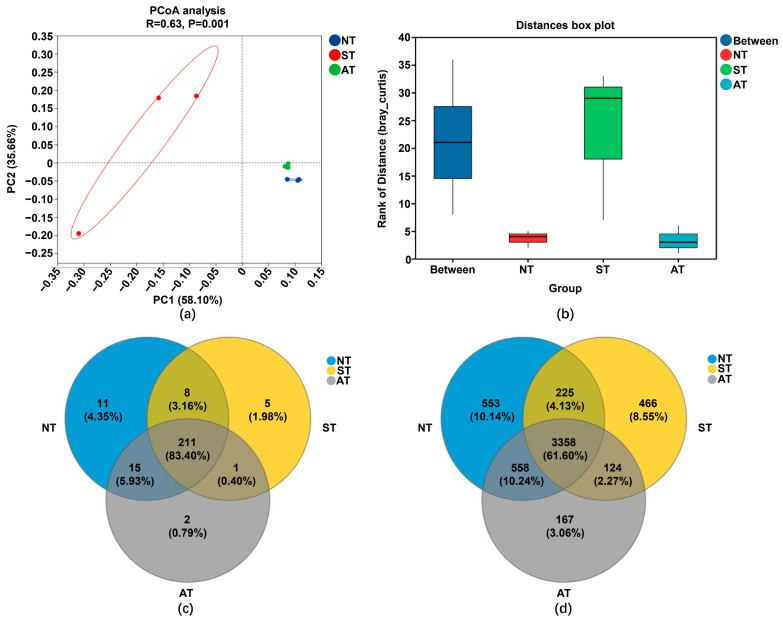
Beta diversity and Venn diagram analysis of peanut rhizosphere bacterial community structure in different soil types. (**a**) PCoA analysis of peanut rhizosphere bacterial community structure in different soil types. (**b**) ANOSIM analysis of peanut rhizosphere bacterial community structure in different soil types. (**c**) Venn diagram analysis of peanut rhizosphere bacterial community structure at the phylum level in peanut rhizosphere soil in different soil types. (**d**) Venn diagram analysis of peanut rhizosphere bacterial community structure at the genus level in peanut rhizosphere soil in different soil types.

Beta diversity was also performed to reflect the dissimilarity in species composition across diverse communities or samples. A significant difference in variance could be observed between soil groups in principal co-ordinates analysis (PCoA): the bacterial communities of NT and AT cluster together in a single quadrant, exhibiting a proximity and a distinct separation from ST, suggesting a clear difference of the community structures between ST and NT (Figure 5a). A deeper dive into between-group differences reveals that inter-group variations are notably greater than intra-group variations in the analysis of similarities (ANOSIM; Figure 5b). The divergence between NT and ST is more pronounced than that between NT and AT, suggesting that saline–alkali soil exerts a more profound influence on the peanut rhizosphere bacterial community compared to acidic soil. Further Venn diagram analysis illuminated that the composition overlap between AT and NT soil was 89.33% and 71.84% at the phylum and genus levels, respectively, and 86.56% and 65.73% between ST and NT at the corresponding levels (Figure 5c,d). This underscores that the composition similarity between AT and NT is greater than that between ST and NT, aligning with the finding of beta diversity analysis, which indicates that saline–alkali soil exerts a more substantial impact on the bacterial community compared to acidic soil.

### 2.6. Diversified Soil Types Differentially Regulated the Peanut Rhizosphere Bacterial Community Structure

**Figure 6:**
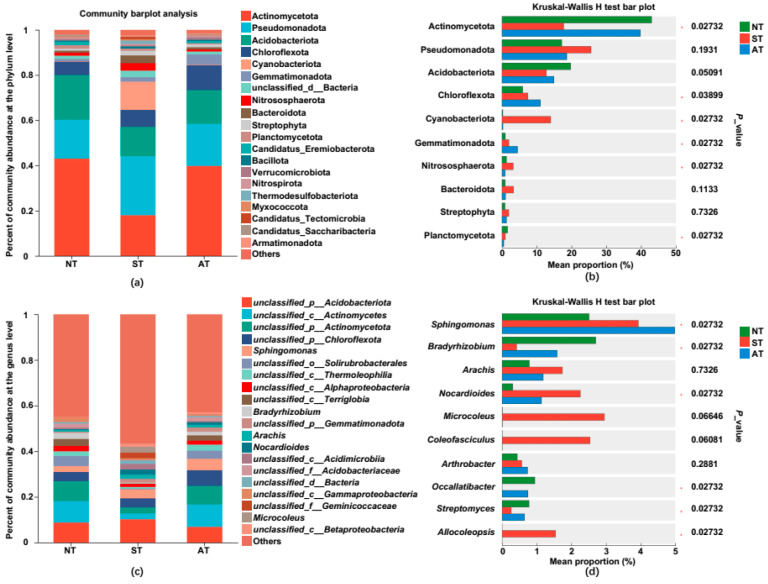
Bacterial community structure of three rhizosphere soil types at the phylum and genus levels. (**a**) Percent of taxa at the phylum level in diverse rhizosphere soils. The relative abundance of each taxon was calculated by averaging the abundances of three duplicates in each soil group. (**b**) Kruskal–Walls H test bar plot of richness at the phylum level. (**c**) Percent of taxa at the genus level in diverse rhizosphere soils. (**d**) Kruskal–Walls H test bar plot of richness at the genus level.

An in-depth analysis was performed on the peanut rhizosphere bacterial communities at the phylum, class, order, family, and genus levels in three distinct soil types. Our findings revealed that the dominant phyla across all three soil types were similar, encompassing Actinobacteriota, Pseudomonadota, Acidobacteriota, and Chloroflexi. However, the abundance of different bacteria varied considerably (Figure 6a). When compared to NT soil, the abundance of Actinobacteriota and Acidobacteriota decreased in both ST and AT soils, with Actinobacteriota experiencing a decline by 58.30% and 7.76% and Acidobacteriota decreasing by 34.30% and 24.32%, respectively. Pseudomonadota accounted for 26.14% and 18.63% of the total bacterial abundance in ST and AT soil, respectively, marking an increase of 8.94% and 1.43% compared to NT soil. The abundance of Chloroflexi rose by 26.68% and 85.23% in ST and AT soil, respectively. Additionally, the abundance of Cyanobacteriota in ST soil was notably higher, reaching 12.44%, compared to NT soil (Figure 6a). Kruskal–Wallis rank-sum test further confirmed that Actinobacteriota was the dominant phylum in AT and NT soil, whereas Pseudomonadota emerged as the more dominant phylum in ST soil (Figure 6b).

At the class level, Actinomycetes and Alphaproteobacteria emerged as the dominant classes in both NT and AT soils, while Alphaproteobacteria and Cyanophyceae dominated in ST soil (Figure S1). When examining the order level, Terriglobales and Solirubrobacterales exhibited lower abundances in AT and ST compared to NT, whereas Sphingomonadales displayed an increase (Figure S2). Furthermore, the top 20 most abundant families across the three treatments were selected and analyzed, revealing the notable disparities in the relative abundance of family-level bacterial composition between ST and NT, while relatively minor differences were observed between AT and NT (Figure S3a). The dominating families primarily encompassed various unnamed species alongside Sphingomonadaceae (Figure S3a). In comparison to NT, the abundance of Acidobacteriaceae declined by 99.03% and 30.52% in ST and AT, respectively, and Nitrososphaeraceae exhibited reductions of 80.56% and 39.24%, respectively (Figure S3a). Moreover, Kruskal–Wallis rank-sum test indicated that Acidobacteriaceae was the dominant family in NT, whereas Sphingomonadaceae prevailed in both ST and AT (Figure S3b).

Statistical analysis and comparison of rhizosphere bacterial communities across diverse soil types revealed that unnamed genera accounted for a high proportion, and *Sphingomonas* was the primary dominant genus (Figure 6c). *Bradyrhizobium*, *Occallatibacter*, and *Streptomyces* stood out as the dominant genera in NT soil. *Bradyrhizobium* decreased by 84.50% in ST and 41.33% in AT. *Occallatibacter* showed a substantial decrease of 99.99% in ST and 21.28% in AT, and *Streptomyces* reduced by 66.67% in ST and 17.95% in AT, respectively. Notably, in ST soil, the abundance of *Sphingobacteriota* increased by 56.57% compared to NT (Figure 6d). These results underscored the significant differences in bacterial communities among the various soil types. The beneficial phyla Actinobacteriota and Acidobacteriota, along with the genera *Bradyrhizobium*, *Occallatibacter*, and *Streptomyces*, exhibited more pronounced decreases in saline–alkali soil compared to acidic soil, which may lead to the creation of an adverse micro-environment and further inhibit peanut growth.

### 2.7. Linear Discriminant Analysis Effect Size (LEfSe) Analysis

Through linear discriminant analysis (LDA) and LEfSe cladogram analysis, we meticulously compared and classified the bacterial communities in peanut rhizosphere soil of various soil types, adhering to the established biomarker screening criteria (LDA score > 3.5). Statistical analysis was conducted across multiple taxonomic levels, from the phylum to the genus, to elucidate these differences. The results revealed that Terriglobia, encompassing its class, order, family, and genus (Terriglobia, Terriglobales, Acidobacteriaceae, and *Occallatibacter*), was notably enriched in NT soil. In contrast, Cyanobacteriota emerged as the dominant phylum in ST soil, with significant enrichment observed for Betaproteobacteria, spanning its class, order, and family (Betaproteobacteria, Burkholderiales, and Sphaerotilaceae). Meanwhile, Chloroflexota, extending from its phylum to genus (Chloroflexota, Ktedonobacteria, Ktedonobacterales, Ktedonobacteraceae, and *Ktedonobacter*), was both dominant and significantly enriched in AT soil (Figure S4). LEfSe analysis with a greater abundance of differential bacteria in ST soil corroborated the findings of the bacterial community structure analysis (Figure 6), suggesting that saline–alkali soil exerts a more substantial influence on the bacterial community in peanut rhizosphere soil compared to acidic soil.

### 2.8. Environmental Factor Analysis

**Figure 7:**
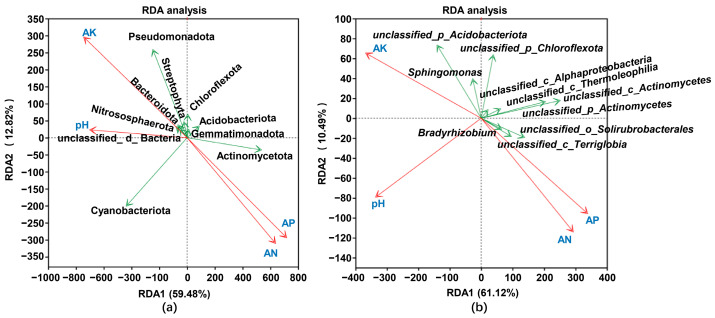
Correlation between peanut rhizosphere bacterial communities and environmental factors in different soil types. (**a**) Correlation between peanut rhizosphere bacterial communities at the phylum level and environmental factors in different soil types. (**b**) Correlation between peanut rhizosphere bacterial communities at the genus level and environmental factors in different soil types. The red arrow represents environmental factors, while the green arrow points to different bacterial phyla in (**a**) or genera in (**b**).

We employed redundancy analysis (RDA) to explore the relationship between peanut rhizosphere bacterial communities and environmental factors across various soil types. The results showed that, at the phylum level, the RDA1 and RDA2 axes collectively explained 72.30% of the variation. Specifically, Actinomycetota exhibited a positive correlation with available phosphorus (AP) and alkali-hydrolyzable nitrogen (AN) while showing a negative correlation with available potassium (AK) and pH. Cyanobacteriota displayed a positive correlation with pH and AN, with no correlation with AK and AP. Pseudomonadota was positively correlated with AK and pH, whereas Acidobacteriota demonstrated a negative correlation with pH (Figure 7a). At the genus level, the RDA1 and RDA2 axes explained 71.61% of the observed variation. *Sphingomonas* was positively correlated with AK but negatively correlated with AP and AN. Conversely, *Bradyrhizobium* was positively correlated with AP and AN while demonstrating a negative correlation with AK and pH (Figure 7b). The RDA analysis shows a close relationship between different bacterial groups and soil physicochemical properties.

### 2.9. KEGG Functional Enrichment Analysis of Microorganisms

**Figure 8:**
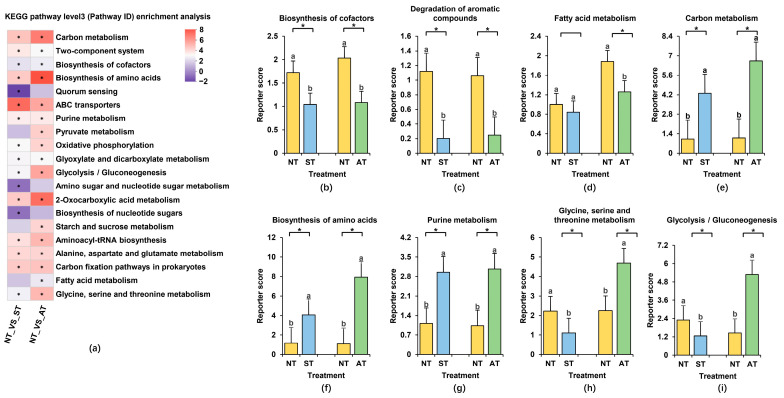
KEGG pathway enrichment of differential metabolites in peanut rhizosphere soil of different soil types. (**a**) Heatmap of KEGG metabolic pathways in peanut rhizosphere soil of different soil types. (**b**–**i**) Bar chart of enrichment analysis for biosynthesis of cofactors (**b**); degradation of aromatic compounds (**c**); fatty acid metabolism (**d**); carbon metabolism (**e**); biosynthesis of amino acids (**f**); purine metabolism (**g**); glycine, serine, and threonine metabolism (**h**); and glycolysis/gluconeogenesis (**i**). The * symbol and different lowercase letters indicate significant difference at *p* < 0.05 compared to NT.

Metabolic pathway enrichment analysis, drawing upon the KEGG database for differential metabolites, provides valuable insights into the biological functions, metabolic processes of soil microorganisms, and their intricate interactions with the environment. Consequently, we delved into the top 20 most significantly enriched pathways in each sample group for a comprehensive understanding. Our findings revealed that both ST and AT had impacts on bacterial metabolites (Figure 8a). Specifically, pathways such as biosynthesis of cofactors, quorum sensing, aminobenzoate degradation, amino sugar and nucleotide sugar metabolism, biosynthesis of nucleotide sugars, degradation of aromatic compounds, and fatty acid metabolism showed decreased abundance in both ST and AT (Figure 8a–d). Conversely, pathways like carbon metabolism, biosynthesis of amino acids, and purine metabolism were notably enriched in both ST and AT (Figure 8e–g). Beyond these shared pathways in ST and AT, there were distinct variations in several pathways via further analysis. For instance, pathways including glycine, serine, and threonine metabolism as well as glycolysis/gluconeogenesis decreased in ST but increased in AT (Figure 8h,i). This underscores that the metabolic pathways in ST and AT, while sharing similarities, also possess unique characteristics and differences. The relatively fewer enriched metabolic pathways in ST compared to AT may be one of the contributing factors to its lower yield. Crucial metabolic activities such as biosynthesis of cofactors, degradation of aromatic compounds, and fatty acid metabolism exhibited reductions in both ST and AT (Figure 8b–d), combining with the lower yield per plant in these soils (Figure 2a), suggesting that these down-regulated metabolic pathways may play a pivotal role in influencing peanut growth and yield.

## 3. Discussion

Excellent soil properties greatly facilitate the growth and development of peanuts. Factors such as soil texture, pH levels, nutrient contents, moisture levels, salt concentration, heavy metal contents, and bacterial communities are all important influencing factors exerting crucial roles in this process. The effects of varied soil conditions on peanuts primarily become evident in their growth environment, root development, and, ultimately, yield. Excessively acidic soil can disrupt the soil’s aggregate structure, causing compaction that hinders root respiration and nutrient absorption [4,33,34]. Additionally, aluminum ions in acidic soil can be toxic to peanut roots, slowing their growth and resulting in shallower, shorter roots, which in turn affects the growth and development of the aboveground parts [35]. Saline–alkali soil, on the other hand, can cause heavy metal elements such as calcium and magnesium in the soil to form insoluble precipitates with phosphates and trace elements, blocking soil pores and affecting soil aeration and water permeability. This leads to peanut yellowing, wilting, and other disease phenomena; increases the incidence of pests and diseases; and hinders the growth of peanut roots and the overall growth and development [6,7,8,36]. Our study confirms that while acidic soil affects multiple agronomic traits and yield components of peanuts, saline–alkali soil has a greater impact on these traits (Figure 1 and Figure 2). This study indicates that lower soil enzyme activity, poorer soil quality, and less favorable rhizosphere micro-environment in ST may, to some extent, lead to a decrease in peanut yield compared to AT. Further investigation into other potential yield-influencing factors is also necessary.

Rhizosphere bacterial communities play an important role in vital activities such as nitrogen fixation and phosphorus solubilization for plants [3]. *Sphingomonas*, as a dominant genus in saline–alkali soil, can significantly improve peanut salt tolerance and stress resistance [25]. Consistent with previous reports, the dominant genus *Sphingomonas* in saline–alkali soil in this study may also play an important role in alleviating the inhibition of peanut growth by saline–alkali soil, which needs further study. Previous studies have found that the number of Cyanobacteria and Acidobacteria mainly increases under saline–alkali soil conditions, while Actinobacteria and Chloroflexi decrease after salt treatment [37]. This is similar to the results of our study, where the abundance of Cyanobacteria was significantly higher in ST than that in NT, and the abundances of Actinobacteriota and Chloroflexi were significantly lower than those in NT (Figure 6a,b). We speculate that the substantially enriched Cyanobacteria in saline–alkali soil may help enhance peanut salt tolerance [38]. We hypothesize that both salt and pH in ST are important factors affecting soil microorganisms, especially salt [39,40], which may have a greater impact on the bacterial community than AT in our study (Figure 6). In terms of bacterial community composition at the genus level, ST and AT also have some similar regulatory mechanisms, such as the relative abundances of *Bradyrhizobium* and *Streptomyces* being significantly lower than those in NT and *Sphingomonas* higher (Figure 6c,d). *Bradyrhizobium* not only stimulates the formation of nitrogen-fixing nodules but also secretes organic acids and enzymes to release more nutrients from the soils, promoting the growth of other beneficial microorganisms in the soils [41,42,43]. The organic acids and carbon dioxide produced by *Streptomyces* can help form large aggregates of soil particles, thereby improving soil structure and enhancing soil aeration and water retention [44,45]. *Sphingomonas* can degrade organic metal compounds in the soils, remediate environmental pollution, and produce beneficial plant hormones, thereby alleviating plant growth under salt stress [46,47,48]. Therefore, we speculate that the reduction in the abundances of beneficial rhizobacteria *Bradyrhizobium* and *Streptomyces* in ST and AT, especially in ST, may impair their abilities to improve soil quality, release nutrients, and decompose organic pollutants, ultimately affecting the growth status of peanuts and leading to reduced yields.

Soil enzymes are crucial in soil nutrient cycling, serving as indicators of soil fertility and environmental quality [49]. Invertase activity is linked to soil carbon cycling, microbial biomass, and intensity of soil respiration, and catalase can indicate the soil microbial life activities to some extent [50]. Soil urease can mineralize organic nitrogen into plant-available inorganic forms, while soil phosphatase plays an important role in hydrolyzing soil organic phosphate into inorganic phosphate. These processes exert important roles in soil nitrogen and phosphorus cycles, contributing to the improvement of soil quality and fertility [51,52]. The decline in the average activities of phosphatase, urease, and invertase in ST as well as urease and invertase activities in AT can impair the soil quality to some extent (Figure 4), potentially hindering peanut growth and leading to a decrease in yield.

At the metabolic pathway level, both ST and AT are significantly enriched in carbon metabolism and the biosynthesis of amino acids, with a higher degree of enrichment in the AT group than in the ST group (Figure 8a,e,f). These pathways have been shown to play important roles in the basic life-sustaining activities and material metabolism of soil microorganisms as well as their resistance to low-carbon-to-nitrogen-ratio stress [53]. Therefore, we speculate that these metabolic pathways may contribute to the enhancement of peanut stress resistance in these low- to medium-yield areas. Additionally, the biosynthesis of cofactors and the degradation of aromatic compounds are significantly reduced in both ST and AT (Figure 8b,c). The biosynthesis of cofactors can effectively maintain the activity of soil microorganisms, promoting their growth and reproduction. A reduction in their abundance may lead to decreased bacterial activity, which is unfavorable for improving soil fertility and structure [54]. The degradation of aromatic compounds and the intermediate products produced have been shown to help restore and maintain ecological balance, reduce toxicity to soil and crops, and promote soil fertility and crop growth [55,56]. Fatty acid metabolism is significantly reduced in the AT group (Figure 8d), and a decrease in its abundance can lead to a reduction in fatty acid content, a component of bacterial membranes. This may weaken the bacteria’s ability to cope with environmental stress, which is adverse for plant growth and stress resistance [57,58]. Based on the earlier research findings, pathways such as the degradation of aromatic compounds, the biosynthesis of cofactors, and fatty acid metabolism may be involved in the degradation of harmful soil substances, soil remediation, soil fertility improvement, and plant stress tolerance. The reduction in these metabolic pathways in saline–alkali and acidic soils may diminish soil fertility, elevate levels of harmful soil substances, or impair plant stress tolerance, ultimately influencing the plant’s growth performance and resulting in decreased peanut yield. The fewer enriched metabolic pathways in the ST group may explain its more significant impact on peanut yield (Figure 8). In the peanut production in low- to medium-yield fields, we may increase the peanut yield by implementing soil-management practices such as inoculating the beneficial rhizobacteria, improving the soil quality, and enhancing soil enzyme activities.

By conducting a comprehensive analysis of the intricate relationships among soil physicochemical factors, soil enzyme activities, rhizosphere bacterial community structure, and peanut yield, this study offers a significant theoretical foundation and practical guidelines for enhancing peanut yield in low- to medium-yield fields, particularly those with acidic and saline–alkali soils. Optimizing rhizosphere micro-environment and increasing soil nutrient contents are crucial to the effectiveness of cropping practices in acidic and saline–alkali soils in the future.

## 4. Materials and Methods

### 4.1. Experiment Site and Design

The pot experiment was conducted at a growth chamber of Laixi Experimental Station of Shandong Peanut Research Institute (36°89′ N, 120°52′ E). Soil samples were collected from the 1–10 mm depth of experimental fields in Laixi (36°89′ N, 120°52′ E), Dongying (37°26′ N, 118°34′ E), and Weihai (37°30′ N, 122°7′ E), representing NT, ST, and AT, respectively. The soil indicators were comprehensively measured, revealing the following: NT had an AN of 67.24 mg/kg, AP of 87.50 mg/kg, AK of 87.50 mg/kg, and a balanced pH of 6.31. Conversely, ST exhibited the lower AN level of 27.86 mg/kg, AP of 5.23 mg/kg, AK of 115 mg/kg, a higher pH of 8.06, and a salt content of 2.44‰. AT displayed an AN of 49.46 mg/kg, AP of 61.60 mg/kg, AK of 64.50 mg/kg, and an acidic pH of 4.91. Approximately 25 kg of three soil types, after being sieved for uniformity, were placed into plastic pots of identical specifications, featuring a bottom diameter of 36 cm and a height of 25 cm. The peanut cultivar cv. Huayu 25 was chosen as the experimental material, with full and uniform seeds planted on 13 May 2023. Eight seeds were sown per pot at a depth of 4 cm, and after emergence, only four seedlings with consistent growth were retained. The peanuts were cultivated under optimal growth conditions (16 h light/8 h dark, 300 mmol m^−2^ s^−1^, 28 °C/18 °C (day/night)) in a growth chamber [59]. Water was added every two days to maintain the soil water content at 75% of field capacity using the weighing method until the harvest time. Each treatment group was represented by three pots. The experimental pots were arranged as a randomized block design. The harvest was conducted on 28 September 2023, with each pot individually assessed.

### 4.2. Sample Collection

When the peanuts reached full maturity, rhizosphere soil samples were meticulously gathered from each treatment group: The pots were emptied entirely, and any soil loosely clinging to the roots was gently shaken off. A sterilized brush was employed to meticulously brush away the soil adhering tightly to the roots, thereby isolating the rhizosphere soil. Peanut debris (including roots), stones, and other impurities were removed during the collection process [60]. Rhizosphere soil from each individual peanut plant within each treatment was painstakingly collected and thoroughly blended together. The amalgamated soil samples were then carefully placed into 15 mL sterile centrifuge tubes. These tubes were promptly frozen in liquid nitrogen to ensure preservation and stored at −80 °C.

### 4.3. Soil Physicochemical Properties and Enzyme Activity Assays

Soil pH was measured in a soil–water suspension (ratio of 1:5) using a pH meter (FE20, Mettler-Toledo Instruments, Zurich, Switzerland), as described by previous study [61]. The soil AN was extracted by using the alkaline hydrolysis diffusion method titrated with standard sulfuric acid solution (5 mmol/L). Soil AP was determined by sodium bicarbonate (NaHCO_3_) spectrophotometry (absorbance at 880 nm, UV-759S, SHIMADZU, Kyoto, Japan) [62]. Soil AK was measured by ammonium acetate flame photometry methods [63]. Soil invertase activity was determined using the 3,5-dinitrosalicylic acid colorimetric spectrophotometry (absorbance at 508 nm, UV-759S, SHIMADZU, Kyoto, Japan), whereas urease activity was determined using sodium phenolate and sodium hypochlorite spectrophotometry (absorbance at 578 nm, UV-759S, SHIMADZU, Kyoto, Japan). Soil catalase activity was assessed by mixing 2 g of fresh rhizosphere soil with 40 mL of distilled water and 5 mL of 0.3% H_2_O_2_ in a flask. Subsequently, the flask was sealed and shaken at 120 rpm for 20 min, and 5 mL of 1.5 M H_2_SO_4_ was added to terminate the reaction. Finally, the solution was filtrated, and 25 mL of filtrate was titrated using KMnO_4_. Soil acid, neutral, and alkaline phosphatase activity was examined by using Solarbio Soil Acid, Neutral, and Alkaline Phosphatase (S-NP) Activity Assay Kits (BC0140; BC0460; BC0280), respectively [64].

### 4.4. Metagenomic Sequencing and Data Analysis

Soil genomic DNA was extracted by using Mag-Bind^®^ Soil DNA Kit (Omega Bio-tek, Norcross, GA, USA) strictly following the manufacturer’s instructions. The concentration and quality of the extracted DNA were assessed using a NanoDrop2000 spectrophotometer (Thermo Fisher Scientific, Waltham, MA, USA) and agarose gel. Genomic DNA that met the quality standards was fragmented to an average size of approximately 400 bp using the Covaris M220 system (Gene Company Limited, Hong Kong, China), and the paired-end library was meticulously constructed using the NEXTFLEX Rapid DNA-Seq Kit (Bioo Scientific, Austin, TX, USA). Adapters, containing the full complement of sequencing primer hybridization sites, were ligated to the blunt ends of the fragments. Subsequently, paired-end sequencing was conducted on the Illumina NovaSeq platform (Illumina Inc., San Diego, CA, USA) at Majorbio Bio-Pharm Technology Co., Ltd. (Shanghai, China), utilizing the NovaSeq 6000 S4 Reagent Kit v1.5 (300 cycles) following the manufacturer’s instructions (http://www.illumina.com, accessed on 17 January 2024). The software fastp (https://github.com/OpenGene/fastp, version 0.23.0, accessed on 17 February 2024) was used to trim adapter sequences at the 3′ and 5′ ends of the reads for quality control based on a minimum Q score of 20 and a minimum sequence length of 50 bp. Then, the sequences were spliced using FLASH (http://www.cbcb.umd.edu/software/flash, version 1.2.7, accessed on 17 February 2024). The clean reads attained exceptional Q20 (98.33%) and Q30 (94.89%) scores, ensuring reliability in taxonomic classification and functional annotation. High-quality pair-end and single-end reads were retained. Then, the reads were aligned to the host DNA sequences by software BWA (http://bio-bwa.sourceforge.net, version 0.7.9a, accessed on 17 February 2024), and the contaminated reads with high similarity were removed.

Metagenomics data were assembled using MEGAHIT (https://github.com/voutcn/megahit, version 1.1.2, accessed on 17 February 2024). Contigs with a length ≥ 300 bp were selected as the final assembling result, and then, the contigs were used for further gene prediction and annotation. Prodigal/MetaGene (https://metagene.nig.ac.jp/metagene/metagene.html, accessed on 2 March 2024) was used to predict ORFs in the contigs from the assembly results. The predicted ORFs with a length ≥100 bp were retrieved and translated into amino acid sequences using the NCBI translation table (http://www.ncbi.nlm.nih.gov/Taxonomy/taxonomyhome.html/index.cgi?chapter=tgencodes#SG1, accessed on 2 March 2024). A non-redundant gene catalog was constructed using CD-HIT (http://www.bioinformatics.org/cd-hit/, version 4.6.1, accessed on 3 March 2024) with 90% sequence identity and 90% coverage [65]. High-quality reads were aligned to the non-redundant gene catalogs to calculate gene abundance with 95% identity using SOAPaligner (https://anaconda.org/bioconda/soapaligner, version 2.21, accessed on 3 March 2024) [66]. Representative sequences of the non-redundant gene catalog were aligned to the NR database with an e-value cutoff of 1 × 10^−5^ using Diamond (http://www.diamondsearch.org/index.php, version 0.8.35, accessed on 9 March 2024) for taxonomic annotations. Functional profiling was performed using Diamond (https://github.com/bbuchfink/diamond, version 2.0.13, accessed on 9 March 2024) against UniRef90 (https://www.uniprot.org/help/uniref accessed on 16 March 2024) to obtain gene families (alignment length 25 aa, identity 80%, and e-value 1 × 10^−5^). The KEGG annotation was conducted using Diamond (http://www.diamondsearch.org/index.php, version 0.8.35, accessed on 13 March 2024) against the Kyoto Encyclopedia of Genes and Genomes database (https://www.kegg.jp/, accessed on 13 March 2024) with an e-value cutoff of 1 × 10^−5^ [67].

### 4.5. Data Analysis

All calculations and plotting were performed using the Majorbio Cloud Platform. Additionally, significant differences between treatment concentrations for differentially expressed metabolites identified in significantly enriched pathways as well as significant differences between treatments for bacterial taxa at the phylum, class, order, family, and genus levels were determined using one-way analysis of variance (ANOVA; * *p* < 0.05). Data processing and image preparation were conducted using R version 4.0.4, GraphPad Prism version 8.3 (GraphPad Software, San Diego, CA, USA), and SPSS software (SPSS, Inc., Chicago, IL, USA, version 19.0).

## 5. Conclusions

This study revealed complex variations in soil physicochemical properties, enzyme activities, bacterial community structure, and metabolic pathways in the peanut rhizosphere soil across different soil types. Saline–alkali and acidic soils demonstrated significantly lower peanut pod production. Through the in-depth study, poor soil quality, low soil enzyme activity, and unfavorable rhizosphere micro-environment may be among the reasons for the decline in peanut yield in ST and AT. Furthermore, the reduction in metabolic pathways involved in the degradation of soil harmful substances, soil remediation, soil fertility improvement, and plant stress tolerance, such as aromatic compound degradation, cofactor biosynthesis, and fatty acid metabolism, may also increase the detrimental influence on peanut yield in these soils. This study provides crucial insights and practical guidelines for optimizing soil management and enhancing peanut yield, thereby paving the way for further exploration of bacterial–metabolic interactions under various soil types to foster sustainable agriculture.

## Figures and Tables

**Table 1:** Alpha diversity analysis of peanut rhizosphere bacterial community in different soil types.

	Sample	Ace	Shannon	Simpson
Phylum	NT	234.00 ± 1.00 a	1.78 ± 0.04 b	0.28 ± 0.01 a
	ST	202.67 ± 0.58 c	2.14 ± 0.29 a	0.20 ± 0.07 a
	AT	220.00 ± 1.00 b	1.87 ± 0.03 ab	0.24 ± 0.01 a
Class	NT	439.33 ± 10.07 a	2.73 ± 0.03 a	0.12 ± 0.00 a
	ST	375.33 ± 2.89 c	2.95 ± 0.44 a	0.12 ± 0.08 a
	AT	392.67 ± 5.03 b	2.81 ± 0.04 a	0.10 ± 0.01 a
Order	NT	814.67 ± 27.79 a	3.63 ± 0.02 a	0.05 ± 0.00 a
	ST	674.67 ± 15.04 c	3.82 ± 0.31 a	0.05 ± 0.02 a
	AT	720.00 ± 7.55 b	3.62 ± 0.01 a	0.05 ± 0.00 a
Family	NT	1543.67 ± 65.43 a	3.94 ± 0.02 a	0.04 ± 0.00 a
	ST	1256.00 ± 40.51 c	4.23 ± 0.32 a	0.04 ± 0.02 a
	AT	1353.67 ± 10.07 b	3.94 ± 0.01 a	0.04 ± 0.00 a
Genus	NT	4087.33 ± 100.11 a	4.39 ± 0.02 b	0.04 ± 0.00 a
	ST	3599.33 ± 136.36 b	4.69 ± 0.23 a	0.03 ± 0.01 a
	AT	3723.00 ± 53.84 b	4.34 ± 0.03 b	0.04 ± 0.00 a

Note: Different lowercase letters in the same column indicate significant difference at *p* < 0.05 among the treatments.

## Data Availability

The metagenomic data have been deposited in the China National Center for Bioinformation Biological Project Library with valid accession number PRJCA036119 (https://ngdc.cncb.ac.cn/bioproject/browse/PRJCA036119, accessed on 13 February 2025).

## Supplementary Materials

The following supporting information can be downloaded at https://www.mdpi.com/article/10.3390/plants14081169/s1, Table S1: Summary of metagenomic reads from peanut rhizosphere soil of different soil types; Table S2: Statistics and prediction of metagenomic sequence assembly, gene number, and annotation in peanut rhizosphere soil of different soil types; Figure S1: Bacterial community structure of three rhizosphere soil types at the class level; Figure S2: Bacterial community structure of three rhizosphere soil types at the order level; Figure S3: Bacterial community structure of three rhizosphere soil types at the family level; Figure S4: LEfSe multi-level species hierarchical tree diagram.

## Abbreviations

NT	Neutral soil
ST	Saline–alkali soil
AT	Acidic soil
ORFs	Open reading frames
PCoA	Principal coordinates analysis
ANOSIM	Analysis of similarities
LEfSe	Linear discriminant analysis effect size
LDA	Linear discriminant analysis
RDA	Redundancy analysis
AN	Alkali-hydrolyzable nitrogen
AP	Available phosphorus
AK	Available potassium
