# Supplementary material for: Diversified Soil Types Differentially Regulated the Peanut (Arachis hydropoaea L.) Growth and Rhizosphere Bacterial Community Structure
Source: Plants (Basel). 2025 Apr 9;14(8):1169. doi: 10.3390/plants14081169 (PMC12030640; doi:10.3390/plants14081169)
Supplement: Supplementary file 1 [file plants-14-01169-s001.zip › plants-3554843-supplementary.pdf]

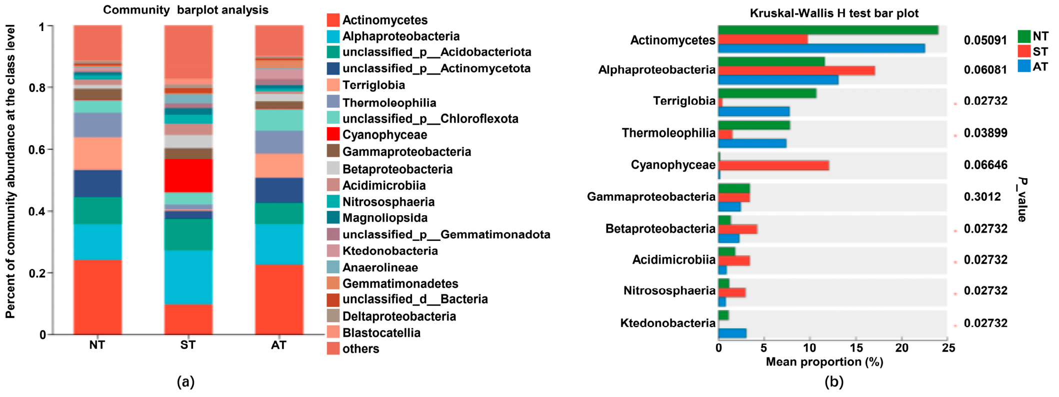

Figure S1. Bacterial community structure of three rhizosphere soil types at the class level
(a) Percent of taxa at the class level in diverse rhizosphere soils. The relative abundance of each taxon was calculated by averaging the abundances of three duplicates in each soil group. **(b)** Kruskal-Walls H test bar plot of richness at the class level.

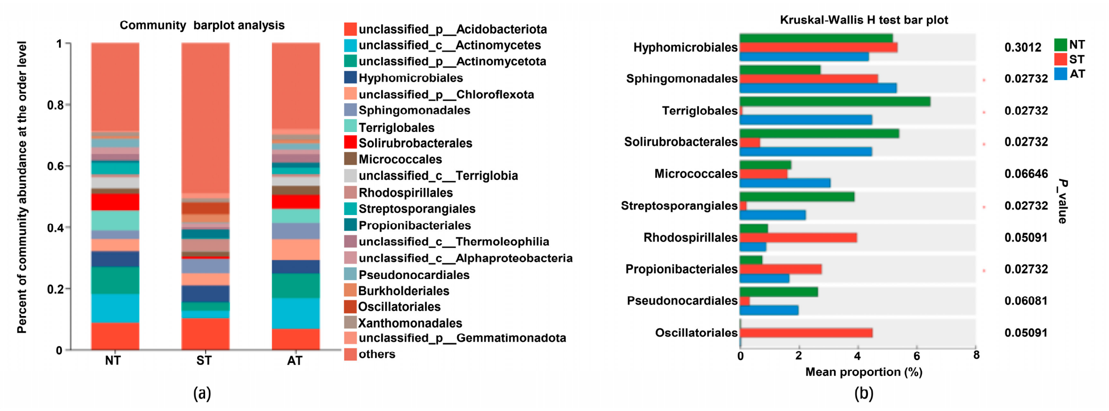

Figure S2. Bacterial community structure of three rhizosphere soil types at the order level
(a) Percent of taxa at the order level in diverse rhizosphere soils. The relative abundance of each taxon was calculated by averaging the abundances of three duplicates in each soil group. **(b)** Kruskal-Walls H test bar plot of richness at the order level.

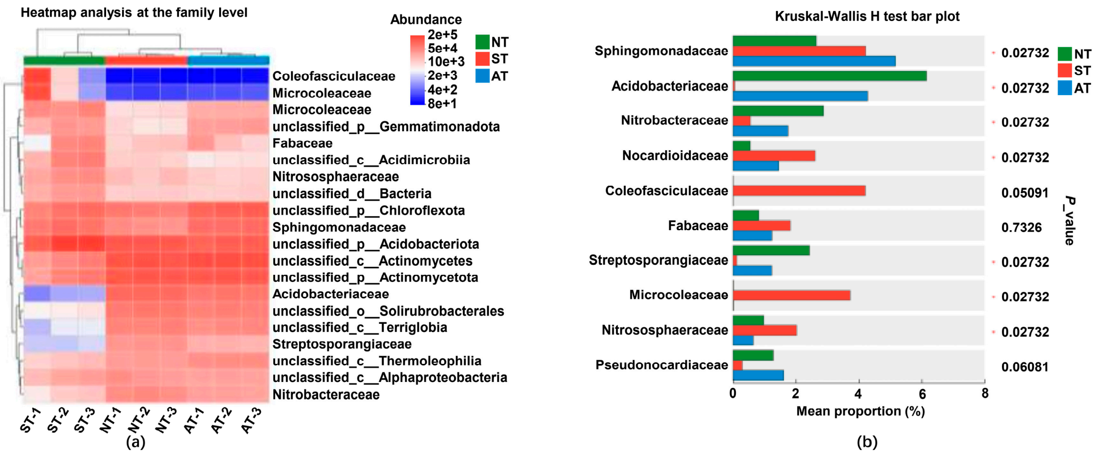

Figure S3. Bacterial community structure of three rhizosphere soil types at the family level
(a) Percent of taxa at the family level in diverse rhizosphere soils. The relative abundance of each taxon was calculated by averaging the abundances of three duplicates in each soil group. **(b)** Kruskal-Walls H test bar plot of richness at the family level.

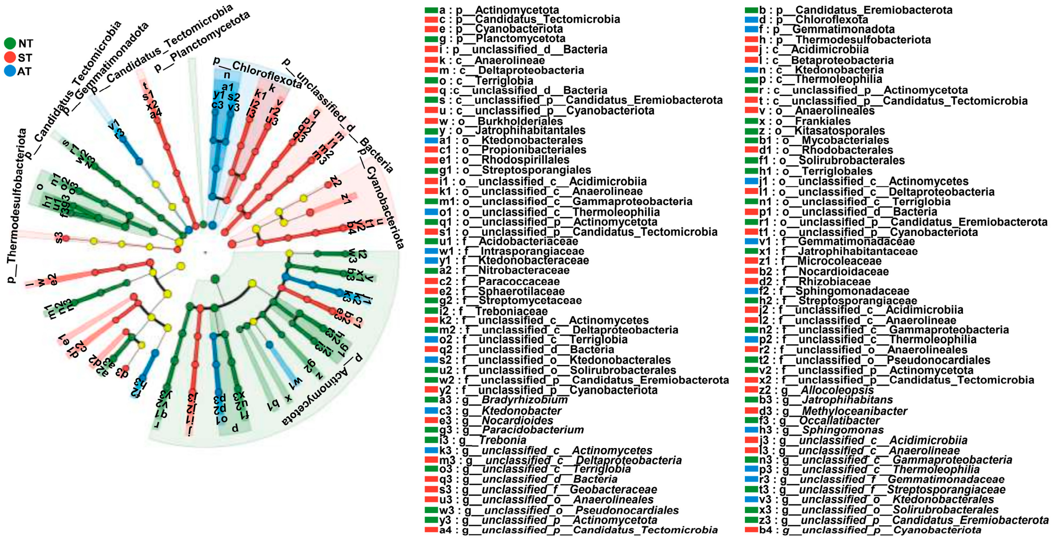

Figure S4. LEfSe multi-level species hierarchical tree diagram

Table S1. Summary of metagenomic reads from peanut rhizosphere soil of different soil types

Sample	Raw reads	Raw base (Mbp)	Clean reads	Clean base (Mbp)	Percent in raw reads (%)	Percent in raw bases (%)
NT-1	44138322	6664.89	43678554	6576.86	98.96	98.68
NT-2	43794954	6613.04	43284660	6517.89	98.83	98.56
NT-3	44940172	6785.97	44509060	6704.23	99.04	98.8
ST-1	47052690	7104.96	46558230	7010.31	98.95	98.67
ST-2	43422364	6556.78	42984168	6473.34	98.99	98.73
ST-3	48911372	7385.62	48428684	7292.56	99.01	98.74
AT-1	42504242	6418.14	42065092	6334.08	98.97	98.69
AT-2	46878726	7078.69	46400486	6980.84	98.98	98.62
AT-3	49506126	7475.43	49020364	7378.26	99.02	98.7

Table S2. Statistics and prediction of metagenomic sequence assembly, gene number, and annotation in peanut rhizosphere soil of different soil types

Sample	Contigs	Contigs bases (Mbp)	N50 (bp)	N90 (bp)	ORFs	Total Length (bp)	Average Length (bp)	Unigenes number
NT-1	485709	323.57	666	353	633662	282724860	446.18	1317599
NT-2	392671	261.46	677	354	510848	228026244	446.37	1278956
NT-3	530239	347.21	655	352	690648	305766588	442.72	1346301
ST-1	317765	158.68	489	331	357336	136082886	380.83	597767
ST-2	252746	154.1	592	341	307818	133542126	433.83	527097
ST-3	331742	167.99	499	332	374374	144186705	385.14	635460

AT-1	401160	244.1	606	345	493824	212156229	429.62	1156861
AT-2	486056	289.8	589	343	605396	258199773	426.5	1256291
AT-3	516011	319	616	347	656686	284784051	433.67	1275646
